# Term Spontaneous Heterotopic Pregnancy (Abdominal and Intrauterine): A Case Report

**DOI:** 10.30476/BEAT.2021.86588.1153

**Published:** 2021-10

**Authors:** Mozhde Momtahan, Maryam Kasraeean, Azam Faraji, Shaghayegh Moradi-Alamdarloo, Mina Moosaie

**Affiliations:** 1 *Department of Obstetrics and Gynecology, School of Medicine, Shiraz University of Medical Sciences, Shiraz, Iran*; 2 *Maternal-fetal medicine Research Center, Shiraz University of Medical Sciences, Shiraz, Iran*; 3 *Student research committee, Shiraz University of Medical Sciences, Shiraz, Iran*

**Keywords:** Heterotopic pregnancy, Abdominal pregnancy, Twin pregnancy, Term pregnancy

## Abstract

Spontaneous heterotopic pregnancy is a potentially life-threatening condition rarely considered when a patient with an intrauterine pregnancy is asymptomatic or presents with complaints such as abdominal pain. An advanced abdominal pregnancy is even more unusual as the form of the ectopic component outside the context of assisted reproduction and is difficult in diagnosis with very few cases reported in the literature. We report such a case in a 31-year-old primigravida with heterotopic pregnancy which is a fetus in the uterine cavity and the other in the abdominal cavity. Her pregnancy was initially misdiagnosed and managed as a di-amniotic di-chorionic gestation. The correct diagnosis was only made after term delivery of the intrauterine pregnancy. The patient was complicated with severe bleeding which led to disseminated intravascular coagulopathy and massive transfusion. Two other operations were imposed on the patient because of bleeding. The clinical risk factor for ectopic pregnancy was only previous pelvic inflammatory disease in this woman.

## Introduction

Heterotopic pregnancy is an intrauterine acceptable pregnancy and ectopic pregnancy at the same time in a pregnant woman. The ectopic one can be a cervical pregnancy, interstitial, tubal or abdominal pregnancy [1]. Symptoms like abdominal pain or spotting are the patient complaints and it is often found in sonography, but there are cases that have been missed in diagnosis and reported with hemorrhagic shock [2]. These symptoms cannot differentiate the diagnosis between variants of ectopic pregnancies like: abdominal, cervical or tubal.

The heterotopic pregnancy risk factors are an increasing usage of assisted reproductive technology, previous tubal surgery and hydro-salpinx [3]. 

Therefore, we present a case of heterotopic pregnancy for publishing after obtaining informed consent in whom underwent operation and post-operation care in Hafez and Nemazee hospitals affiliated to Shiraz University of Medical Sciences. She was missed in diagnosis and had perinatal care like twin pregnancy and dichorion diamnion. The main diagnosis was made during the operation. Although we know that abdominal pregnancy is a kind of ectopic pregnancies that was found in the literature but there is not a report like this case which took care like twin pregnancy till term. 

## Case Presentation

**Fig. 1:**
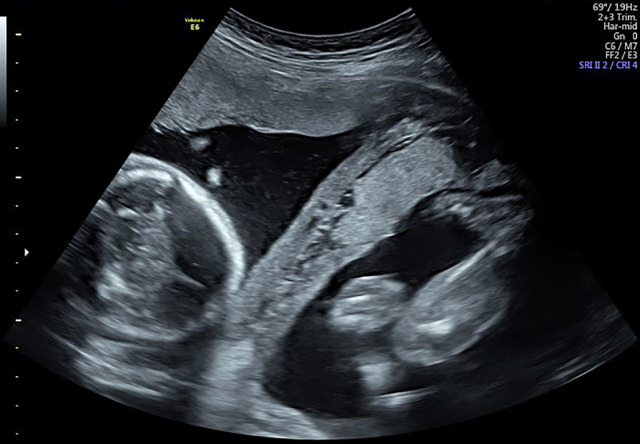
A 36 weeks gestational age, dichorionic diamniotic twin pregnancy presented with labor pain

A 31-year-old primigravida female at 36 weeks and 4 days of gestational age with dichorionic diamniotic twin pregnancy presented by labor pain (Figure 1). Presenting part of the fetuses was breech-vertex. The woman had no history of infertility or taking ovulation induction. The last ultra-sonographic examination was one week prior to admission and reported no abnormality; fetus A had anterior placenta and the other had posterior placenta. This examination reported a thick membrane between the two fetuses by ultrasound.

The patient was prepared for cesarean section with Pfannensteil and Kerr incision; the first baby was delivered which was a boy the with Apgar score 8 in first minute and 10 in the fifth minutes, weighing the 2240 gram. No other fetus was in uterine cavity, therefore, the uterus was lifted through the incision after placental delivery and an intact amniotic sac was in the abdominal cavity. The skin incision extended to T shape and a girl was delivered with Apgar score 8 in the first minute and 10 in fifth minutes; her weight was 2200 gram.

The abdominal baby placenta was adherent to the right adnexa, cecum and the pelvic cavity right side which was traumatized while the baby was being delivered. This caused severe bleeding in the abdominopelvic cavity. Four bags of uncrossed packed cell were transfused at first immediately which the general surgeon rushed for help and clamped the aorta for 20 minutes to help control the bleeders. Finally, they packed the cavity with 3 long gases. Totally, 8 bags of packed cell, 14 bags of platelet, 14 bags of fresh frozen plasma, and 10 bags of cryoprecipitate were transfused.

The patient was transferred to the Intensive care unit (ICU) post-operation while she was intubated. She went under reoperation in 7 hours after the first operation because of the low mean arterial pressure which not responding to blood products and volume transfusion and abdominal distention. The bleeding control and repack were done and then she was transferred to the ICU. Laboratory findings were hemoglobin: 7.5, platelet: 44000, fibrinogen level: 89 after operation; therefore, 4 bags of packed cell, 7 bags of platelet, 3 bags of fresh frozen plasma, and 10 bags of cryoprecipitate were transfused. Twenty-four hours’ post-operation laboratory data were as bellow after these transfusions: Hemoglobin: 10, platelets: 100000, fibrinogen: 130, and creatinine: 1.4.

The patient was again transferred to the operation room to remove the pack after 48 hours of repack. Therefore, the patient underwent conservative management two days after the last operation and she was discharged with two healthy babies in the fifth day post-cesarean section.

In the history of this patient, she only reported two times taking pelvic inflammatory disease’s treatment due to malodor vaginal discharge in several months before pregnancy. 

## Discussion

This study was reported a term heterotopic pregnancy case which was diagnosed post-operation and complicated with massive transfusion, multiple operations and disseminated intravascular coagulopathy. 

Heterotopic pregnancy is a condition of threatening pregnant woman in early gestational ages, but in this case, diagnosis delay in twin healthy babies till near term ended and she was discharged well after several days of the hardworking of the medical teams.

In the literature, the most heterotopic pregnancies are an intrauterine good pregnancy and a tubal or interstitial pregnancy that are diagnosed in early pregnancy and the ectopic one terminated with medical or surgical treatments [4, 5].

Saving intrauterine pregnancy is a main concern when a heterotopic pregnancy is diagnosed for a pregnant woman as Chen *et al*., [6] reported 144 cases of heterotopic pregnancy and 85.6% live birth rate after surgery, but all of ectopic pregnancies were treated in the first trimester. 

Abdominal pregnancy is a very rare kind of ectopic pregnancy which several articles have been reported on this disease in fetus’s term. Abdominal pregnancy has two types of primary abdominal pregnancy or secondary after tubal abortion [7, 8]. All of these reported patients were missed to diagnose abdominal pregnancy. The most important finding in this case was reporting the uterine wall between two fetuses as a thick membrane for twin pregnancy (dichorion diamnion).

## Conclusion

Ultrasonic examination is the first step to diagnose heterotopic pregnancies, therefore, careful extra-uterine examination is necessary and finding a good intrauterine pregnancy is not cause for finalizing examination. Any doubt in diagnosis should be confirmed with other imaging modalities. The surgeon should consider this pathologic pregnancy in cesarean section and should be careful not to traumatize the placenta.
